# Enabling oxygen-controlled microfluidic cultures for spatiotemporal microbial single-cell analysis

**DOI:** 10.3389/fmicb.2023.1198170

**Published:** 2023-06-20

**Authors:** Keitaro Kasahara, Markus Leygeber, Johannes Seiffarth, Karina Ruzaeva, Thomas Drepper, Katharina Nöh, Dietrich Kohlheyer

**Affiliations:** ^1^IBG-1: Biotechnology, Institute of Bio- and Geosciences, Forschungszentrum Jülich GmbH, Jülich, Germany; ^2^Computational Systems Biotechnology (AVT.CSB), RWTH Aachen University, Aachen, Germany; ^3^Aachen Institute for Advanced Study in Computational Engineering Science (AICES), RWTH Aachen University, Aachen, Germany; ^4^Institute of Molecular Enzyme Technology, Heinrich Heine University Düsseldorf, Forschungszentrum Jülich GmbH, Jülich, Germany

**Keywords:** microfluidic, oxygen control, FLIM, microbial single-cell analysis, PDMS, RTDP, automated image analysis

## Abstract

Microfluidic cultivation devices that facilitate O_2_ control enable unique studies of the complex interplay between environmental O_2_ availability and microbial physiology at the single-cell level. Therefore, microbial single-cell analysis based on time-lapse microscopy is typically used to resolve microbial behavior at the single-cell level with spatiotemporal resolution. Time-lapse imaging then provides large image-data stacks that can be efficiently analyzed by deep learning analysis techniques, providing new insights into microbiology. This knowledge gain justifies the additional and often laborious microfluidic experiments. Obviously, the integration of on-chip O_2_ measurement and control during the already complex microfluidic cultivation, and the development of image analysis tools, can be a challenging endeavor. A comprehensive experimental approach to allow spatiotemporal single-cell analysis of living microorganisms under controlled O_2_ availability is presented here. To this end, a gas-permeable polydimethylsiloxane microfluidic cultivation chip and a low-cost 3D-printed mini-incubator were successfully used to control O_2_ availability inside microfluidic growth chambers during time-lapse microscopy. Dissolved O_2_ was monitored by imaging the fluorescence lifetime of the O_2_-sensitive dye RTDP using FLIM microscopy. The acquired image-data stacks from biological experiments containing phase contrast and fluorescence intensity data were analyzed using in-house developed and open-source image-analysis tools. The resulting oxygen concentration could be dynamically controlled between 0% and 100%. The system was experimentally tested by culturing and analyzing an *E. coli* strain expressing green fluorescent protein as an indirect intracellular oxygen indicator. The presented system allows for innovative microbiological research on microorganisms and microbial ecology with single-cell resolution.

## Introduction

In nature, microbial cells are constantly exposed to dynamically changing conditions in inhabited microenvironments. Consequently, cells respond to environmental changes and adapt to new environments and ecological niches. In addition to nutrient availability, temperature, pH, and osmolarity, the level of dissolved O_2_ belongs to the most important environmental factors. Oxygen is affecting microbial growth, metabolism as well as the physiological state and behavior of cells. According to their ability to utilize O_2_ as a primary electron acceptor during respiration, microbes can be assigned either to the group of aerobes or anaerobes. While obligate aerobes and anaerobes essentially need either sufficient O_2_ supply or strict O_2_ limitation for growth, facultative aerobic and anaerobic microbes can cope with O_2_ fluctuations by altering their metabolism (e.g., via a change from aerobic respiration to anaerobic respiration or fermentation processes) (André et al., 2021; Long et al., 2021).

Besides these general aspects of microbial metabolism, O_2_ further influences many other physiological processes and morphological properties of bacteria and microbial consortia, including oxidative stress (via the formation of reactive O_2_ species as by-products of O_2_ metabolism), iron homeostasis (via spontaneous or enzymatic oxidation of soluble ferrous to insoluble and thus difficult to access ferric iron), biofilm formation, and infection (Cabiscol et al., 2000; Miethke and Marahiel, 2007; André et al., 2021; Mart'ın-Rodr'ıguez, 2022). In addition, O_2_ limitation is an ecological force that strongly shapes the global nitrogen and carbon cycle or the development and composition of microbiomes (for example, microbial communities in O_2_ minimum zones or the gut microbiota) (Byndloss et al., 2018; Long et al., 2021). Furthermore, limited O_2_ availability has immense relevance for diverse biotechnological and biomedical applications.

Microfluidic cultivation devices, which allow precise environmental control, have enormous potential to mimic natural microenvironments and study the effects of dynamic O_2_ levels on microbial single-cell physiology, populations and communities. In the last decade, experimental approaches combining time-lapse microscopy and microfluidics have been widely used to spatially and temporally resolve the behavior of single microbes and populations, for example, in terms of microbial growth (Lindemann et al., 2019), phenotypic population heterogeneity (Mustafi et al., 2014), and cellular interactions (Burmeister et al., 2018; Mukherjee and Bassler, 2019; Burmeister and Grünberger, 2020; Ren et al., 2022; Schito et al., 2022). Clearly, image-based single-cell analysis can provide new insights into the exchange of nutrients, conjugative transfer of plasmids, quorum sensing, as well as host-pathogen relations when studying cellular interactions. Interaction studies benefit greatly from innovative microfluidics and time-lapse imaging, as these single-cell approaches can provide complementary and novel data that are not accessible with population-based laboratory-scale methods.

The required microfluidic chips typically contain channels and chambers with a height corresponding to the diameter of the cultured cells. This height restriction nicely results in monolayer growth of micropopulations, which is ideal for subsequent image-analysis tasks such as cell segmentation, tracking and more. Cells can be grown and studied under constant culture conditions simply by continuously perfusing the culture media through the supply channels. Even dynamic conditions can be elegantly generated by changing the chemical composition of the culture media, when controlling the position of multiple parallel laminar-flow streams in the cultivation area (Täuber et al., 2020, 2022). The cultivation temperature is typically kept constant by placing the microfluidic setup inside the microscope incubator.

The control of O_2_ in microfluidics has been demonstrated for a variety of applications (Skolimowski et al., 2010; Abaci et al., 2012; Lam et al., 2018). However, only few concepts are easily transferable to microbial single-cell analysis when focusing on usability. The availability of O_2_ can be continuously adjusted by implementing additional equipment or structures, for example by implementing a small gas incubator around the microfluidic chip. A microfluidic (de)oxygenator structure containing additional gas channels might also be implemented into the chip device, facilitating diffusive gas exchange between the fluid channel (containing the culture medium) and two adjacent gas channels (delivering the desired gas flow), via the gas-permeable chip material (Funamoto et al., 2012). The silicone polydimethylsiloxane (PDMS) is the most commonly used chip material due to its high gas permeability (Chang et al., 2014; Koens et al., 2020).

Since microfluidic culture chips contain micrometer-sized growth chambers, conventional sensors cannot be used to measure O_2_, but novel bottom-up approaches have been developed. Among the few O_2_ sensing principles implemented into microfluidics, O_2_-sensitive dyes allow online monitoring of O_2_ and continuous fluid flow at the same time. For example, the fluorescence of the chemical dye tris(2,2'-bipyridyl)dichlororuthenium(II)hexahydrate (RTDP) is quenched in the presence of O_2_ described by the Stern-Volmer equation and can be used as an oxygen indicator. Oxygen-sensitive dyes were widely used for fluorescence intensity-based measurements (Sun et al., 2018; Wang et al., 2018; Liu et al., 2023). Other methods using oxygen-sensitive surface coatings were based on Förster resonance energy transfer (FRET) and ratiometric measurements (Ungerböck et al., 2013). Since the fluorescence lifetime is independent of the dye concentration, fluorescence lifetime imaging (FLIM) provides more robust and accurate O_2_ measurements compared to intensity-based and ratiometric approaches (Gerritsen et al., 1997). By performing FLIM in the frequency domain and in combination with a dedicated microscope camera, O_2_ can be imaged with spatial and temporal resolution during live-cell microscopy on a microfluidic device (Chang et al., 2014; Wu et al., 2019; Hsu et al., 2021).

Although O_2_ control and on-chip sensing have been successfully demonstrated in microfluidic devices already several years ago, these attempts were made using relatively large cultivation channels for mammalian cells and were less suitable for microbial single-cell analysis (Wu et al., 2018). In addition, it remains a challenge to efficiently extract quantitative cellular properties from thousands of microscopy images acquired during time-lapse imaging. Integrating automated image analysis into the experimental approach could improve the efficiency and accuracy of data extraction. In order to more systematically investigate the effect of O_2_ levels in (micro)environments on microbial behavior, a comprehensive experimental platform is required. The setup should allow microbial cultures and single-cell analysis in defined O_2_ environments. Finally, subsequent automated image analysis is essential to derive new information from statistically reliable large data sets.

This methodology paper describes a comprehensive experimental setup for the spatiotemporal single-cell analysis of microbes under well-controlled environments including O_2_ control. Our approach consists of three main experimental parts, namely: (i) on-chip cultivation and online control of O_2_ in-flow, (ii) online O_2_ monitoring by fluorescence lifetime imaging of the oxygen-sensitive dye RTDP, and (iii) time-lapse microscopy for automated image acquisition and subsequent deep learning (DL)-based image analysis.

For this purpose, the microfluidic chip was placed inside a low-cost 3D-printed mini-incubator, ensuring reliable gas exchange and precise on-chip O_2_ control. A (de)oxygenator structure was included in the chip design, although it is optional for the present work. O_2_ was monitored on-chip by imaging the fluorescence lifetime of the O_2_-sensitive dye RTDP and calculating the corresponding O_2_ concentrations. The capability of our system for on-chip O_2_ control and sensing was evaluated by performing several O_2_ switches, where the amount of O_2_ inside the gas supply was step-wisely increased during FLIM measurement. Microbial cultivation requires small structures of the order of 1 μm and below in height to allow high-resolution optical observation. Therefore, our microfluidic chip contains cultivation chambers with a height of 1 μm. These chambers restrict cell growth to monolayers allowing to study microbiological physiology in a confined O_2_ environment at the single-cell level. As an microbiological application example, *Escherichia coli* MG1655 expressing green fluorescent protein (GFP) as an additional intracellular O_2_ indicator, was grown under aerobic (21% O_2_) and anaerobic (0% O_2_) conditions. The image data acquired from time-lapse microscopy was analyzed using our recently developed automated image-analysis tools, which allow the extraction of cellular properties at the single-cell and population level. The paper is also intended to demonstrate the power of microfluidics to microbiologists interested in using microfluidic systems for their specific research. The developed platform could be used as a general tool for different biological studies.

## Materials and equipment

###  Microfluidic device fabrication

The microfluidic cultivation chip was fabricated following a two-layer soft lithography. Briefly, a 100 mm silicon wafer was subsequently coated with two layers of the photoresist SU-8 (Microchemicals GmbH, Germany) and structured by photolithography. The masks for the photolithography were designed with the layout editor Clewin5 (WieWeb software, The Netherlands) and the generated GDS layout file is available as a Supplementary material.

The fabricated silicon wafer containing the SU-8 structure served as the mold for the following PDMS casting step. 50 g of a PDMS mixture (Sylgard 184 Silicone elastomer kit, Dow Chemical Company, USA) was prepared by thoroughly mixing the monomer base with the crosslinker in a ratio of 10:1, resulting in a homogeneous and opaque highly viscous mixture. This mixture was then degassed in a desiccator at 200 mbar underpressure for 1 h to remove any air bubbles. The resulting fully transparent PDMS mixture was poured over the silicon wafer mounted in a common petri dish. Thermal curing was then performed at 80°C for at least 1 h. The crosslinked PDMS slab was manually peeled from the silicon mold and cut into single chips. Then inlets and outlets for the fluid and the gas flow connections were manually punched using a punching tool (ϕ = 0.75 mm, World Precision Instruments, USA). Single PDMS chips were finally bonded to glass substrates (D263^®^Bio, 39.5 mm × 34.5 mm × 0.175 mm, Schott AG, Germany) after an O_2_ plasma treatment (Femto Plasma Cleaner, Diener Electronics, Germany) for 25 s. For full fabrication details, the reader is referred to Gruenberger et al. (2013) and Täuber et al. (2021).

###  Gas and fluid flow control

Mass flow controllers calibrated for two different mass-flow ranges (0.5 - 10 mL/min and 20 - 600 mL/min, red-y, Vögtlin Instruments GmbH, Switzerland) and the gasses O_2_ and N_2_, were used to mix the gas at desired O_2_ and N_2_ ratios (gas supply pressure: *p* = 4 bar). A mini-incubator (50.0 × 82.0 × 18.0 mm) was designed (Solidworks, Dassault Systems, France) and fabricated by 3D printing (Original Prusa i3 MK3S, Prusa Research, Czech Republic) (Supplementary Figures S1A, B). The O_2_ concentration inside the mini-incubator was continuously measured by an inserted laboratory-scale O_2_ sensing probe (VisiFerm mA 120 H3, Hamilton, USA). The 3D CAD and printing files are available as Supplementary material. Fluid flow was controlled using a syringe pump (neMESYS, CETONI, Germany) for the continuous delivery of the sensor solution and the culture medium.

###  Oxygen sensor

For O_2_ sensing inside the microfluidic chip, 5 mg/mL of RTDP solution (Acros Organics, Belgium) dissolved in Milli-Q water (Merck Millipore, USA) was used. The prepared RTDP solution was protected from light and stored inside an anaerobic workbench (DG250, Don Whitley Scientific, UK) until use.

###  Microscopy setup

Microfluidic cultures and O_2_ imaging were performed on an inverted and automated live-cell microscope (Nikon Eclipse Ti-E 2, Nikon, Japan) equipped with a 20 × objective (Plan Apo λ, Nikon, Japan), a 100 × objective (Plan Apo λ Oil, Nikon, Japan), and the perfect focus system (PFS) for focus drift compensation during time-lapse imaging. The setup was equipped with two digital cameras which can be used sequentially during time-lapse imaging. A CMOS camera (DS-Qi2, Nikon, Japan) was installed to the right microscope port to record phase-contrast and wide-field fluorescence images. The fluorescence excitation light was supplied by a SOLA LED (SOLA Light Engine, Lumencor, USA) and a fluorescence filter cube (GFP-3035D, Nikon, Japan).

The special FLIM camera (550 kHz frequency domain, pco.flim, PCO AG, Germany) connected to the modulated excitation laser (445 nm, 100 mW, pco.flim laser, Omicron-Laserage Laserprodukte GmbH, Germany) was mounted to the left microscope port. A customized filter cube with a emission filter = 440/40 (F47-440, AHF analysentechnik AG, Germany), a dichroic mirror = 495LP (F48-495, AHF analysentechnik AG, Germany), and a emission filter = 605/70 (F47-605, AHF analysentechnik AG, Germany) was used for FLIM. Additionally, the microscope setup was surrounded by a temperature incubator (Okolab, Italy), ensuring stable cultivation temperature. A customized chip holder fitting into the motorized X-Y-stage was used to mount the microfluidic chip and the 3D-printed mini-incubator (Supplementary Figures S1C, D). The chip holder CAD layout is available as Supplementary material.

###  Microbial cultures

*E. coli* MG 1655 carrying the vector pVWEx-1-gfpUV and a kanamycin resistance gene, was used for microbial growth experiments. GFP expression in the *E. coli* strain was induced by the addition of 0.1 mM isopropyl β-D-1-thiogalactopyranoside (IPTG, Carl Roth, Germany). Microbial cultures were performed according to the following procedure. The primary preculture was performed in a complex LB medium (Supplementary Table S1). For all subsequent culture steps, defined minimal M9 medium supplemented with 2% glucose (Carl Roth, Germany), 2% casamino acid (VWR, USA), and 50 μg/mL kanamycin (Carl Roth, Germany) was used (Supplementary Table S2). First, a shake flask containing 15 mL of LB medium was inoculated with a single colony from an agar plate and cultured at 37°C and 150 rpm for approximately 8 h. The second preculture containing M9 minimal medium was inoculated from the primary culture, with an initial optical density (OD_600_) value of 0.05, and cultivated overnight at 30°C, 120 rpm. The final shake flask culture was inoculated from the second preculture with initial OD_600_ = 0.3, and cultivated at 30°C and 120 rpm until the culture reached the logarithmic growth phase. Cells derived from the log phase culture were seeded into the growth chambers of the microfluidic chip.

## Methods

###  Technical concept

**Figure 1:**
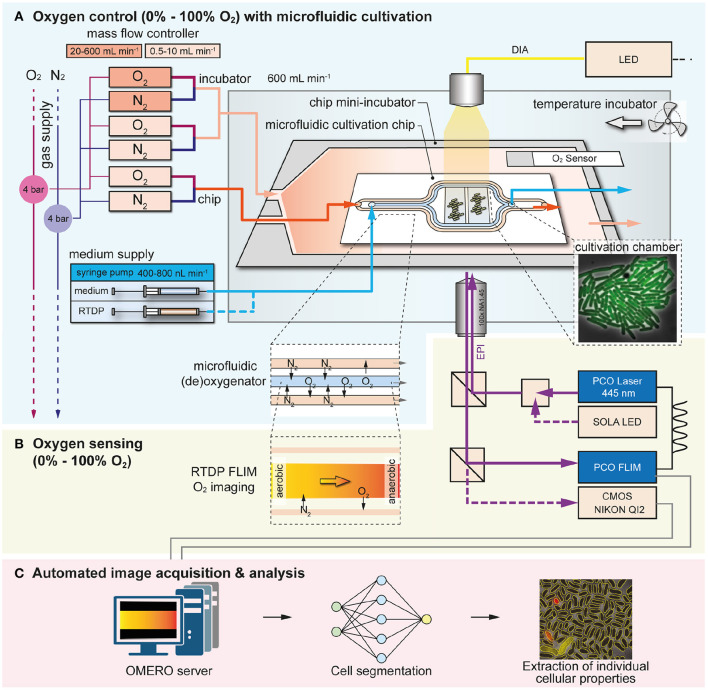
Overview of the microfluidic cultivation system for spatiotemporal microbial single-cell analysis under a controlled O_2_ environments. **(A)** O_2_ control was achieved by using a microfluidic chip with (de)oxygenator structure inside an 3D-printed mini-incubator over the range of 0% - 100%. **(B)** O_2_ sensing with spatial and temporal resolution was implemented by imaging the fluorescence lifetime of the O_2_-sensitive dye RTDP. **(C)** Automated image acquisition and analysis was performed with special software tools.

An experimental system for the control of O_2_ during microbial single-cell analysis has been developed (Figure 1). The system includes: (A) a microfluidic cultivation chip with a (de)oxygenator structure for on-chip O_2_ control placed inside a 3D-printed mini-incubator, (B) online O_2_ sensing by FLIM of the oxygen-sensitive dye RTDP, and (C) and automated image analysis procedure based on DL (Figure 1).

The 3D-printed mini-incubator was required to deliver a continuous gas flow of the desired O_2_/N_2_ composition over the PDMS chip. As the chip material PDMS is highly gas permeable (diffusion coefficient for O_2_ ≈10^−9^ m^2^ s^−1^), it facilitates efficient gas exchange by diffusion across the entire chip surface (Vollmer et al., 2005; Markov et al., 2014). When we operated the chip without the incubator, anaerobic conditions were not possible due to uncontrolled O_2_ influx from the surrounding laboratory atmosphere. Thus, the mini-incubator enables cultures in the absence of O_2_ when 100% N_2_ was supplied continuously and undesired influx was prevented.

Gas flow was controlled using four interconnected mass flow controllers (O_2_ 0.5 - 10 mL/min, O_2_ 20 - 600 mL/min, N_2_ 0.5 -10 mL/min and N_2_ 20 - 600 mL/min) to automatically mix O_2_ and N_2_ in desired ratios prior to the microfluidic chip and mini-incubator. The mini-incubator was operated at a total gas flow of 600 mL/min and a gas flow of 10 mL/min was delivered through the microfluidics. In the microfluidic chip, gas exchange was assisted with the (de)oxygenator structure which facilitates gas diffusion between the fluid and the adjacent gas channels across the PDMS membranes in between, as shown in Figure 1A. The syringe pump was used to deliver either the RTDP solution during on-chip oxygen sensing or the culture medium during single-cell analysis at a flow rate of 400 nL/min. The RTDP-based oxygen sensing was not performed simultaneously with the microbial single-cell analysis, but was used separately to characterize the performance of the device.

Dissolved oxygen in the fluid channel was determined by camera-based fluorescence lifetime imaging of the O_2_-sensitive dye RTDP (Figure 1B). During microbial cultures, growth medium was provided continuously to maintain stable culture conditions. Growth chambers were arranged behind the (de)oxygenator structure to allow sufficient residence time and thus gas-exchange time of the fluid. This allowed the cells to be supplied with (de)oxygenated medium and cultured at the desired O_2_ concentrations. Phase contrast and fluorescence intensity images were obtained using the conventional CMOS camera.

After microfluidic cultivation and continuous image acquisition from multiple chambers, the data can be quite large, making manual handling of the data time-consuming and difficult. Therefore, the acquired image sequences were processed with an in-house developed automated image analysis platform to extract interesting cellular properties such as cell number, area and fluorescence intensity to determine growth rates and cellular activities. Cellular properties can be extracted for each individual cell in the culture chambers, providing detailed information at the single-cell level (Figure 1C).

###  Microfluidic cultivation device

**Figure 2:**
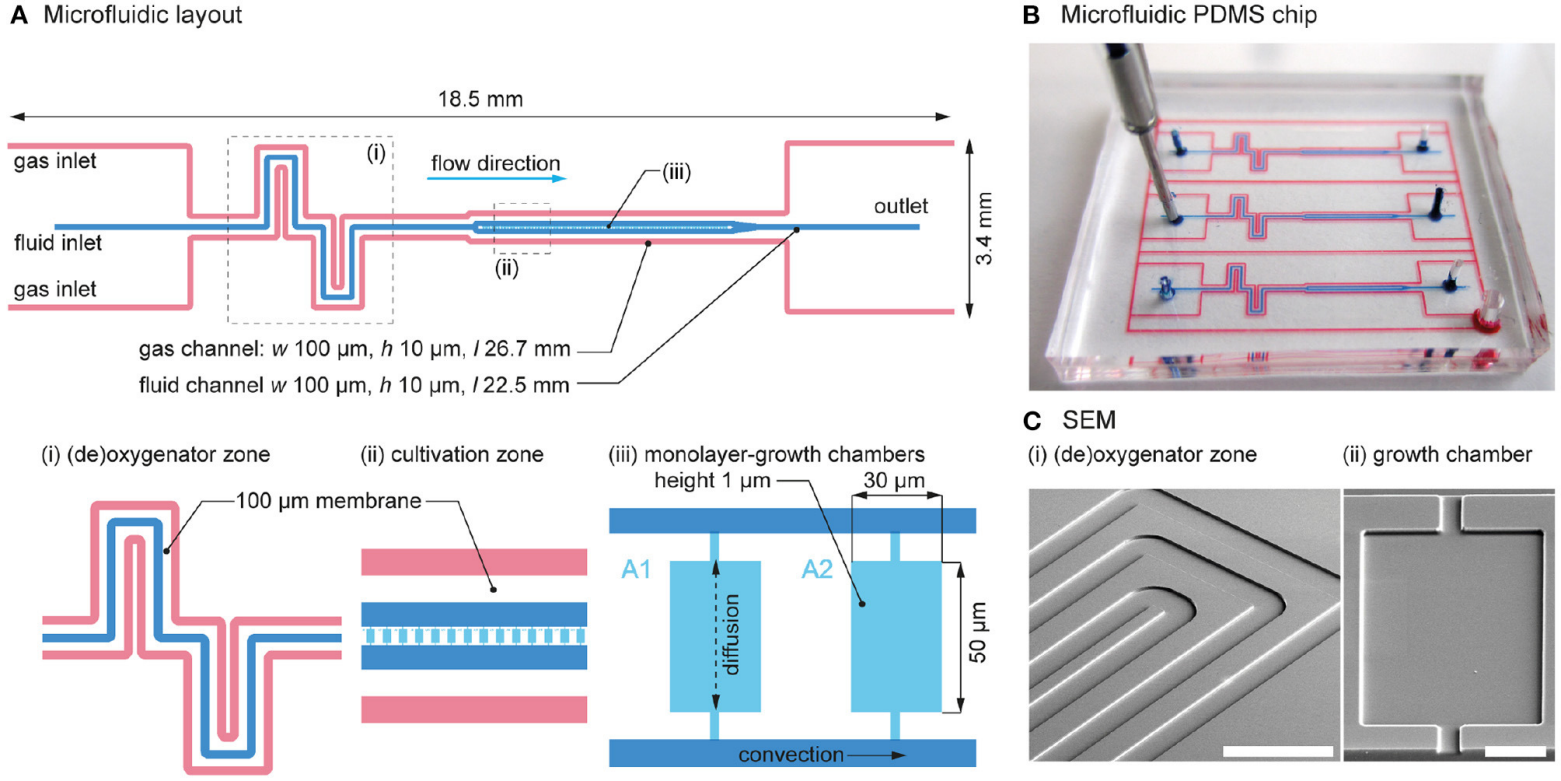
Detailed design of the microfluidic chip. **(A)** Layout of the microfluidic channels, with details of the (i) (de)oxygenator zone, (ii) cultivation zone, and (iii) growth chambers. **(B)** Photograph of the fabricated microfluidic PDMS chip. The chip has the size of a typical postage stamp. For visualization, the gas and fluid channels were colored with red and blue ink. **(C)** SEM images of the microfluidic (i) (de)oxygenator structure (scale bar = 300 μm) and (ii) growth chamber (scale bar = 20 μm).

A new microfluidic chip with the required channel layout was designed to allow the on-chip control and simultaneous cultures (Figure 2A). The fluid channel (blue) is sandwiched between two adjacent gas channels (red). All supply channels are 100 μm wide and 10 μm deep. As shown in Figure 2Ai, ii, each channel comprises (i) a (de)oxygenator zone, followed by (ii) a cultivation zone with 80 cultivation chambers, allowing a statistically reliable number of microbial populations to be imaged during a single experiment.

Between the fluid channel and the adjacent gas channels, a 100 μm thick PDMS wall acts as a gas permeable membrane. The height of a cultivation chamber is 1 μm and the surface area = 50 μm × 30 μm, resulting in a volume of a single cultivation chamber of 1.5 pL. The height of the chamber corresponds to the diameter of a typical *E. coli* cell thereby restricting cell growth to a defined monolayer (Figure 2Aiii). Monolayer cell growth is highly advantageous for subsequent single-cell segmentation tasks on time-lapse images. Depending on the organism to be cultured, the resulting chamber height can be specified by adjusting the photoresist thickness of the spin-coated SU-8 layer during wafer processing. Cultivation chambers are arranged in series and each one is interconnected to two common fluid supply channels. During the cultivation phase, the volume flows in the parallel supply channels are equal. Therefore, no pressure difference and thus no convective flow is induced inside the growth chambers. As a result, mass transfer between the supplied medium and the growth chamber is governed by diffusion and the cells are not affected by shear forces (Westerwalbesloh et al., 2015).

As all gas channels are interconnected, only a single gas inlet and outlet port is required for on-chip gas flow, which results in uniform O_2_ levels across the chip. In addition, all cultivation channels are equipped with adjacent gas channels on either side, creating a homogeneous O_2_ distribution across the cultivation zone.

For illustration purposes, the microfluidic channels of the fabricated chip were visualized by the infusion of red and blue ink, as shown in Figure 2B. The chip contains three parallel culture channels, allowing three simultaneous cultures during a single experiment. The scanning electron microscope images of the fabricated chip show (i) the (de)oxygenator structure with the fluid channel in the center and the adjacent gas channels at the sides, and (ii) the cultivation chamber (i.e., growth chamber) with the rectangular structure and two connections to the fluid supply channels (Figure 2C).

###  On-chip O_2_ sensing by fluorescence lifetime imaging

Prior to the fluorescence lifetime imaging (FLIM), a calibration measurement must be performed with a reference slide containing a fluorescent dye with known and stable lifetime (lifetime = 3.75 ns, UMM-SFG, Starna Scientific, UK). This calibration is required to correctly determine the lifetime from unknown samples, in our case the lifetime of the O_2_-sensitive dye RTDP. The calibration procedure must be performed every time the laser is shut down or operated at different frequency.

The performance of oxygen control was characterized using the RTDP sensor solution. The fluorescence of RTDP is quenched in the presence of O_2_ according to the Stern-Volmer equation (Equation 1), where *K*_*q*_ is the Stern-Volmer constant (the quenching efficiency), τ is the experimentally measured lifetime and τ_0_ is the lifetime in the absence of oxygen.


(1) $$\begin{array}{l} \left[{\text{O}}_{2}\right]=\frac{1}{K_{q}}\left(\frac{\tau_{0}}{\tau }-1\right) \end{array}$$

First, the RTDP solution must be calibrated at two known oxygen concentrations, at 0% O_2_ giving τ_0_ and at, for example, 100% O_2_ to then derive *K*_*q*_. In practice, however, the calibration procedure at 0% O_2_ can be challenging because it requires an anoxic sensor solution. Several procedures have been demonstrated, for example, using a droplet of the sensor surrounded with 100% N_2_ gas, or consuming O_2_ by adding an O_2_ scavenging chemical to the solution (Kalinina et al., 2016; Wu et al., 2019). However, none of these methods were compatible with our approach.

In the present work, the calibration was performed at 0% O_2_ using a gas-tight glass syringe connected to a glass capillary filled with an anoxic RTDP solution to determine τ_0_. The RTDP solution and the syringe were prepared entirely in the anaerobic workbench. Briefly, the solution was gassed with N_2_ for at least 1 h in the anaerobic workbench, then filled into a gas-tight glass syringe (1.0 mL, ILS, Germany) to ensure an anoxic solution (Supplementary Figure S2A). The syringe was connected to a glass capillary (75 μm inner diameter x 375 μm outer diameter x 70 cm length, Molex, USA), which was then manually flushed with the anoxic RTDP solution. Prior to the assembly, 1 cm of the polymer coating was removed in the middle of the capillary using a flame and acetone to prepare an optical detection window for FLIM. The assembled syringe including the capillary was taken out of the anaerobic workbench, fixed above the objective on a suitable specimen holder, the capillary end was connected to a waste container and flushed continuously at 400 nL/min using the syringe pump. Then FLIM was performed to determine τ_0_.

Furthermore, the RTDP solution was calibrated at 100% O_2_ inside the microfluidic chip by using the on-chip microfluidic gas flow combined with the continuous gas flow through the mini-incubator at 100% O_2_ to determine τ_100%_ (Supplementary Figure S2B). Briefly, the fluid channel was connected to a syringe (Omnifix^®^F Solo 1mL, Braun, Germany) via PTFE tubing (0.5 mm inner diameter x 1.6 mm outer diameter, Chromatographie Service GmbH, Germany) and the RTDP solution was delivered at 400 nL/min. Gas flow was maintained at 100% O_2_ for several hours until the oxygen diffusion reached equilibrium and a steady-state lifetime sensor output and τ_100%_ was determined. The obtained τ_0_ and τ_100%_ were used to calculate the *K*_*q*_ value in Equation 1.

Calibration was performed at two different temperatures (*T* = 25°C and 30°C). As evident, the fluorescence lifetime of RTDP is also temperature dependent, thus calibration, measurement and microbial cultures should be performed at the same temperature.

**Figure 3:**
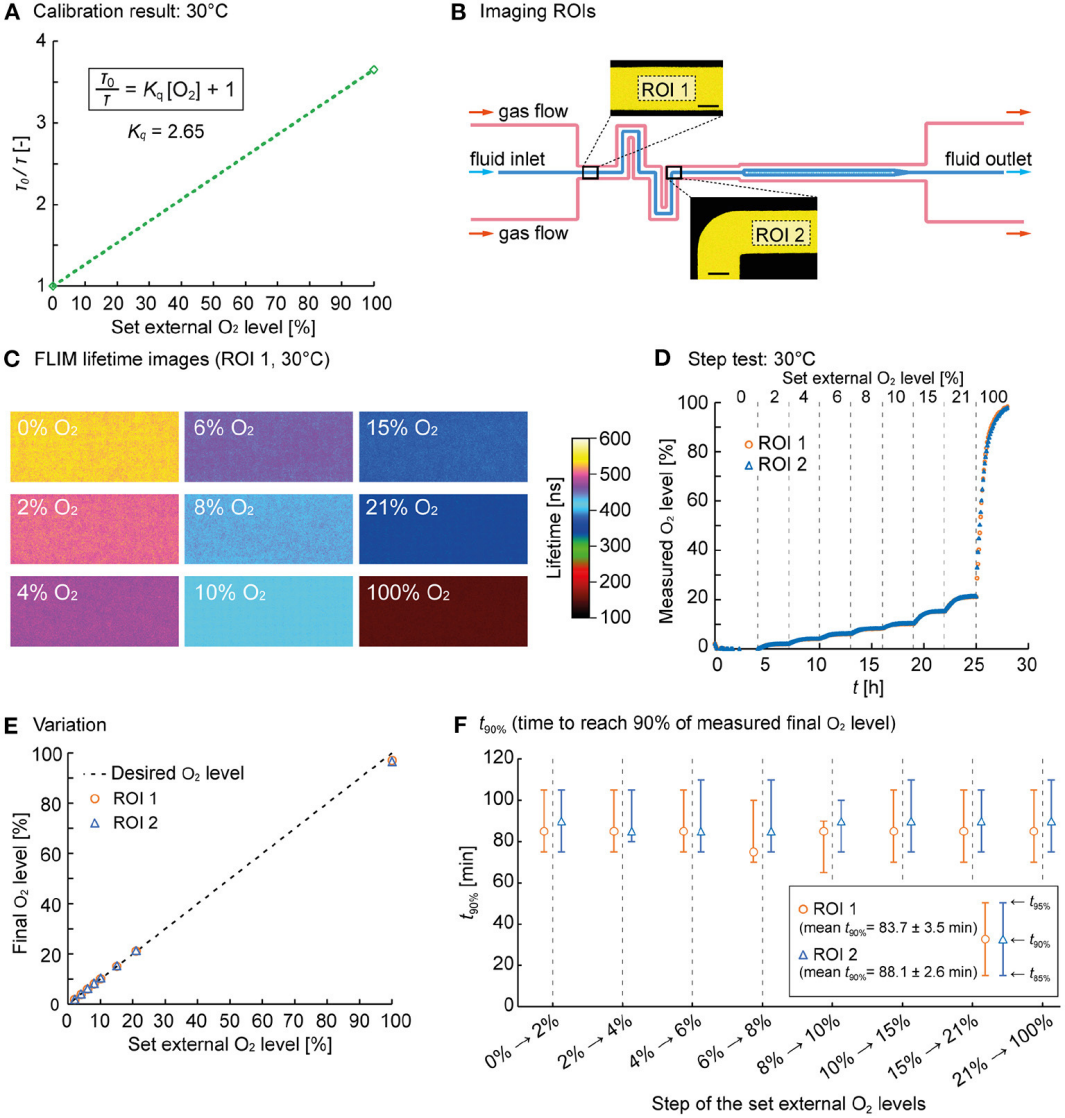
O_2_ control performance characterization. **(A)** Calibration plot of the RTDP sensor solution at 30°C. **(B)** Imaging ROIs 1 and 2 where FLIM of the RTDP oxygen sensor in the fluid channel was performed (scale bars = 50 μm). **(C)** Representative FLIM images obtained at defined O_2_ gas flow. The images were taken at ROI 1 at 30°C. **(D)** O_2_ concentration over time derived from corresponding fluorescence lifetime images at 30°C at ROI 1 and 2. **(E)** Final O_2_ concentration determined by FLIM over the externally applied gas flow. The dotted line shows the optimum. **(F)** Switching time analysis: *t*_90%_ for each step of the set external O_2_ levels. The middle markers indicate *t*_90%_, and the bars indicate *t*_85%_ and *t*_95%_.

The following lifetime values were determined during calibration. At *T* = 25°C, τ_0_ = 573 ns and τ_100%_ = 160 ns (Supplementary Figure S2C), whereas at *T* = 30°C, τ_0_ = 526 ns and τ_100%_ = 144 ns (Figure 3A). Using Equation 1, *K*_*q*_ was calculated as 2.58 at *T* = 25°C and 2.65 at *T* = 30°C. Using these *K*_*q*_ values and Eq. 1, the measured oxygen concentrations could be calculated from the measured fluorescence lifetimes.

###  On-chip oxygen control and sensing - performance characterization

A sequential step test was performed (at *T* = 25°C and 30°C) during O_2_ control to characterize the dynamical performance of our setup. Therefore, a switching sequence was programmed using an in-house written LabView tool controlling the four massflow controllers. The O_2_ / N_2_ ratio of the gas mixture was then changed automatically to generate desired O_2_ concentrations in the fluid flow. The RTDP solution was delivered continuously through the fluid channel, while the gas flow composition through the gas channels and the mini-incubator was set to 0%, 2%, 4%, 6%, 8%, 10%, 15%, 21%, and finally to 100% O_2_ over a period of 30 h. The initial sensing phase at 0% O_2_ was maintained for 4 h to ensure anoxic starting conditions. Then, the gas composition was altered every 3 h. During FLIM the RTDP solution was continuously delivered at 400 nL/min. The O_2_ concentration inside the mini-incubator rapidly followed the desired values and changes were performed with negligible delay as evident from the oxygen sensor readings from the Hamilton probe installed inside the mini-incubator (data not shown).

Time-lapse images of the fluorescence lifetime were taken every 5 min at two successive channel positions, as shown in Figure 3B before and after the (de)oxygenator (ROI 1 and 2). The acquired image sequences from FLIM were processed with Fiji (Schindelin et al., 2012), a distribution of ImageJ (National Institutes of Health, USA) to determine the average lifetime for each image, as these images have a certain noisiness at the level of single pixels. From the representative false-color images determined for each O_2_ concentration at *T* = 30°C (here ROI 1, Figure 3C), it is visible that FLIM delivered lifetime images, revealing homogeneous oxygen availability in the channel. The lifetime decreased with the increasing oxygen concentration, as expected. The time-lapse video of the RTDP solution during oxygen level switching is available as a Supplementary material. Oxygen values were then calculated using Equation 1 and plotted over time in Figure 3D (for *T* = 25°C see Supplementary Figure S2D).

The displayed O_2_ concentration curve shows a response behavior to the concentration switches O_2_ performed. Due to the diffusion-limited oxygen transport, the concentration follows an asymptotic progression until equilibrium and the final value is reached. The final oxygen values in the chip were determined in each equilibrium phase by averaging the measured concentration over the last 1h of each performed step, and plotted over the desired values in Figure 3E (for *T* = 25°C see Supplementary Figure S2E). The graph clearly shows that the final O_2_ concentration reached very comparable values over the whole range from 0% to 100% O_2_.

Finally, we studied the switching performance in terms of time. Therefore, the times required to reach 85%, 90% and 95% of the final O_2_ concentration (*t*_85%_, *t*_90%_, *t*_95%_) were determined and plotted in Figure 3F for *T* = 30°C (for *T* = 25°C see Supplementary Figure S2F). *t*_90%_ is indicated as central marker, whereas *t*_85%_ and *t*_95%_ are represented as the lower and upper bars. The plot show that *t*_90%_ was in the range of 80 to 90 min. *t*_90%_ seems independent of the applied O_2_ step width, as the diffusion time *t* is independent of the concentration difference but depends on the diffusion distance *d* and the specific diffusion coefficient *D* (*t*≈*d*^2^/*D*).

The present system is limited in terms of fast oxygen changes, which can be performed below two hours. This is mainly due to the millimeter-thick PDMS chip. Obviously, the gas exchange can be accelerated by fabricating a thinner PDMS chip to reduce the time required for the gas exchange by diffusion (Polinkovsky et al., 2009; Markov et al., 2014). Our intention here was to develop a relatively simple setup enabling reliable switching and constant culture conditions.

###  Microfluidic cultivation and time-lapse imaging

A cell suspension was prepared using cells harvested from the logarithmic growth phase of the final shake flask culture and diluted to an OD_600_ = 0.35. Cells were then seeded into the microfluidic cultivation chambers by delivering the cell suspension into the fluid channel by applying gentle pressure to the syringe while visually inspecting the seeding performance. After sufficient chambers were seeded with cells, M9 medium was used to rinse residual cells from the supply channels. M9 medium containing 0.1 mM IPTG was then continuously delivered at a flow rate of 400 nL/min. During the cultivation, time-lapse phase contrast and fluorescence images were taken every 5 min. Optimal illumination and camera exposure times have to be experimentally derived for each setup.

In the case of the anaerobic microfluidic cultivation, cell inoculation was performed entirely inside the anaerobic workbench to ensure complete isolation of the cells from O_2_, but without the ability to follow the seeding process by microscopy. After seeding, the glass syringe filled with an anaerobic M9 medium containing 0.1 mM IPTG was connected to the fluid inlet. The entire cultivation setup, including the syringes, the chip and the mini-incubator, was assembled inside the anaerobic workbench. Finally, the prepared setup was removed from the anaerobic workbench and mounted on the microscope stage. The temperature inside the temperature incubator was maintained at 30°C.

###  Automated image analysis

The recorded phase contrast and fluorescence time-lapse image sequences from biological experiments were analyzed to extract the individual intracellular fluorescence intensities. As the image sequences contained more than 50*k* individual cells in total, the image processing was done partly automatically using DL segmentation in Jupyter Notebooks and Python (Seiffarth et al., 2022). First, the acquired time-lapse sequences were cropped into single cultivation chambers, aligned with Fiji, and stored in an OMERO instance (Allan et al., 2012). A pre-trained Omnipose (Cutler et al., 2022) segmentation model was applied to extract the contour of individual cells followed by a border filter eliminating segmentation artifacts closer than 0.5 μm to the image borders. The fluorescence signal for every individual cell is computed by averaging the fluorescence channel intensities inside every segmented cell contour.

After cell segmentation, cell tracking was performed using the activity-prioritized method (Ruzaeva et al., 2022) to explore the distributions of the fluorescence intensities inside every segmented cell contour over the cell generations. The cell lineages were computed using the default parameters of the cell tracker, utilizing the Omnipose segmentation model. Starting from the initially present cell, assigned with generation “zero”, the daughter cells gain one generation after every recorded division event. The analysis was performed for the fixed time point for each data set. The code used for cell segmentation and tracking is available at https://github.com/JuBiotech/Supplement-to-Kasahara-et-al.-2023a.

## Results and discussion

###  Aerobic and anaerobic growth of *E. coli*

#### Population analysis

As a proof of principle application, we investigated the impact of changing O_2_ availability on microbial growth at the single-cell level. *E. coli* expressing green fluorescent protein (GFP) in the presence of the inducer IPTG, was chosen as model system and cultures were performed under aerobic and anaerobic conditions. The final maturation step of the GFP chromophore requires O_2_ present in the cell. As a consequence, GFP fluorescence can only be detected when protein folding was completed at sufficient intracellular O_2_ and the fluorescent protein is fully functional. In the absence of oxygen, however, despite complete protein expression, the chromophore maturation can not be completed and no intracellular fluorescence should occur. However, once maturation has been completed under aerobic conditions, the GFP fluorescence is no longer dependent on the absence or availability of oxygen. Then, the fluorescence intensity is mainly reduced by GFP dilution due to cell division, protein degradation and chromophore bleaching. Thus in the present study, GFP was used as a rudimentary one-way indicator for intracellular O_2_ (in our case, we performed an oxygen increase).

The phase contrast and the fluorescence microscopy images of growing colonies were acquired every 5 min. These time-lapse images were used to determine the dynamics and heterogeneity of intracellular GFP fluorescence in *E. coli* to study O_2_ availability inside the cultivation chambers.

**Figure 4:**
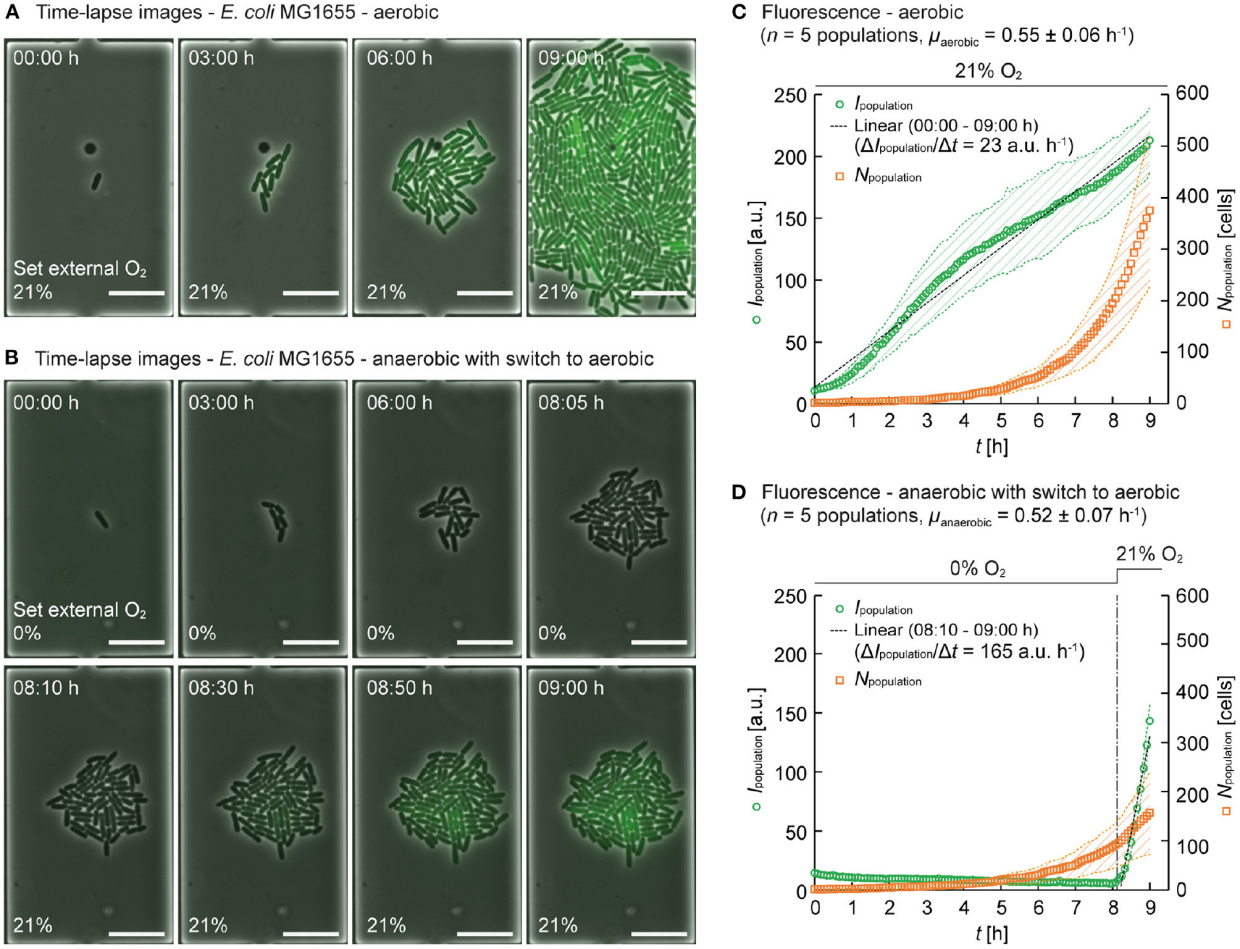
Population based analysis using the model organism *E. coli* MG1655 expressing GFP. **(A)** Time-lapse image series of a *E. coli* MG1655 population cultured at 30°C under aerobic conditions and continuous supply of M9 medium supplemented with 2% glucose (00:00 h ≤ *t* ≤ 09:00 h) (scale bars = 10 μm). **(B)** Time-lapse image series of *E. coli* MG1655 population cultured at 30°C under anaerobic conditions and continuous supply of M9 medium supplemented with 2% glucose (00:00 h ≤ *t* ≤ 08:05 h) with a switch to aerobic conditions (08:10 h ≤ *t* ≤ 09:00 h) (scale bars = 10 μm). **(C)** Mean intensity of GFP fluorescence over population (*I*_population_) and cell number (*N*_population_) of *E. coli* MG1655 populations cultured under aerobic conditions. 1875 cells were analyzed at *t* = 09:00 h. The data are presented as mean ± S.D. denoted by the shaded areas (*n* = 5 individual populations). **(D)** *I*_population_ and *N*_population_ of *E. coli* MG1655 populations cultured under anaerobic conditions with a switch to aerobic conditions. 783 cells were analyzed at *t* = 09:00 h. The data are presented as mean ± S.D. denoted by the shaded areas (*n* = 5 individual populations).

Under aerobic conditions, when infusing 21% O_2_, and the continuous supply of fresh minimal medium, exponential *E. coli* colony growth was observed. An exemplary colony is shown in Figure 4A, where phase contrast and fluorescence intensity images were merged for better visualization. In the presence of oxygen, GFP was expressed in all cells over the entire culture period of 9 hours, as evident from the nearly linear increase of the total fluorescence. The cell numbers (*N*_population_) were counted over the time-lapse images to determine population growth rates. The mean intensity of GFP fluorescence over the population (*I*_population_) was derived to compare fluorescence intensities. For the aerobic cultivation, micropopulations in five independent growth chambers were analyzed. The plot in Figure 4C shows that the populations grew exponentially at the growth rate $\mu_{\text{aerobic}}=0.55\pm 0.06\;{\text{h}}^{-1}$ and the fluorescence *I*_population_ increased nearly linear due to the continuous expression of fully matured GFP. The slope of the approximate line, Δ*I*_population_/Δ*t*, was calculated as 23 a.u. h^−1^ between 00:00 h ≤ *t* ≤ 09:00 h.

Under anaerobic conditions, when infusing 100% N_2_, surprisingly we still observed comparable exponential growth, but as expected no intracellular GFP fluorescence was detectable for the first 8 h, as shown in Figure 4B. After 8 h of culture time, a switch in the gas supply was performed, delivering 21% O_2_ (and 79% N_2_) to the microfluidic culture instead, thereby creating aerobic conditions. Again, five independent micro populations of the anaerobic culture were analyzed (Figure 4D). Under anaerobic conditions, the population growth rate was $\mu_{\text{anaerobic}}=0.52\pm 0.07\;{\text{h}}^{-1}$ (00:00 h ≤ *t* ≤ 08:05 h), and the fluorescence *I*_population_ stayed low and negligible fluorescence was detected. Then, immediately after the switch from anaerobic conditions to aerobic conditions, *I*_population_ increased 7-fold faster and reached comparable values compared to non-switch conditions ($\Delta I_{\text{population}}/\Delta t=165\text{ a}.\text{u}.\;{\text{h}}^{-1}$ between 08:10 h ≤ *t* ≤ 09:00 h).

This 7-fold rapid increase in fluorescence of *I*_population_ after the switch to aerobic conditions can be explained as follows. During the primary anaerobic cultivation phase, GFP expression was performed, but intracellular oxygen was not available (or too low) to complete the chromophore maturation process. Incompletely produced GFP did not emit fluorescence. After switching to 21% O_2_, intracellular oxygen became available and the pending chromophore maturation could be finally completed. In addition, newly expressed GFP might have contributed to the increase in fluorescence. The minimum concentration of O_2_ required for the GFP chromophore maturation was reported to be 0.1 ppm (Hansen et al., 2001). Therefore, we speculate that we can operate our system at concentrations as low as 0.1 ppm O_2_, however, our fluorescence detection limit should be further considered in that context.

Since *E. coli* is a facultative anaerobic organism, and it can continue to grow in the absence of oxygen using fermentation or anaerobic respiration. Interestingly, we found comparable growth rates under both aerobic and anaerobic conditions. It has been reported that *E. coli* grows more slowly in the absence of oxygen (Stolpera et al., 2010), but we believe that the overall growth conditions within the microfluidic growth chambers are superior to conventional cultivation methods, which typically involve batch cultivation. We have previously reported that continuous perfusion of media allows for constant amounts of substrate, no accumulation of inhibitory by-products and therefore cells can exhibit faster growth rates throughout the culture period (Grünberger et al., 2013).

#### Single-cell analysis

Time-lapse imaging includes spatiotemporally resolved data of single-cells. Population-based analysis as shown in the previous paragraph delivered growth rates based on the number of segmented cells. However, tracking of single microbes over subsequent time-lapse images is a more challenging task, especially if the temporal resolution is too low to follow distinct family lineages. This becomes even more difficult if all cells within the population have identical optical appearance. Therefore, we applied the activity-prioritized cell tracking method (Ruzaeva et al., 2022) to explore single-cell fluorescence over the cell generations.

This method enabled us to derive the mean intensity of GFP fluorescence (*I*_cell_) from segmented cells as well as their corresponding generation number (number of cell divisions observed). Fast-growing cells resulted in high generation numbers and slow-growing cells resulted in lower generation numbers. For our analysis, one exemplary micro population was selected from the aerobic and the anaerobic culture, respectively. Three consecutive time points (*t* = 08:00 h, 08:30 h and 09:00 h) were then chosen to visualize our data analysis.

**Figure 5:**
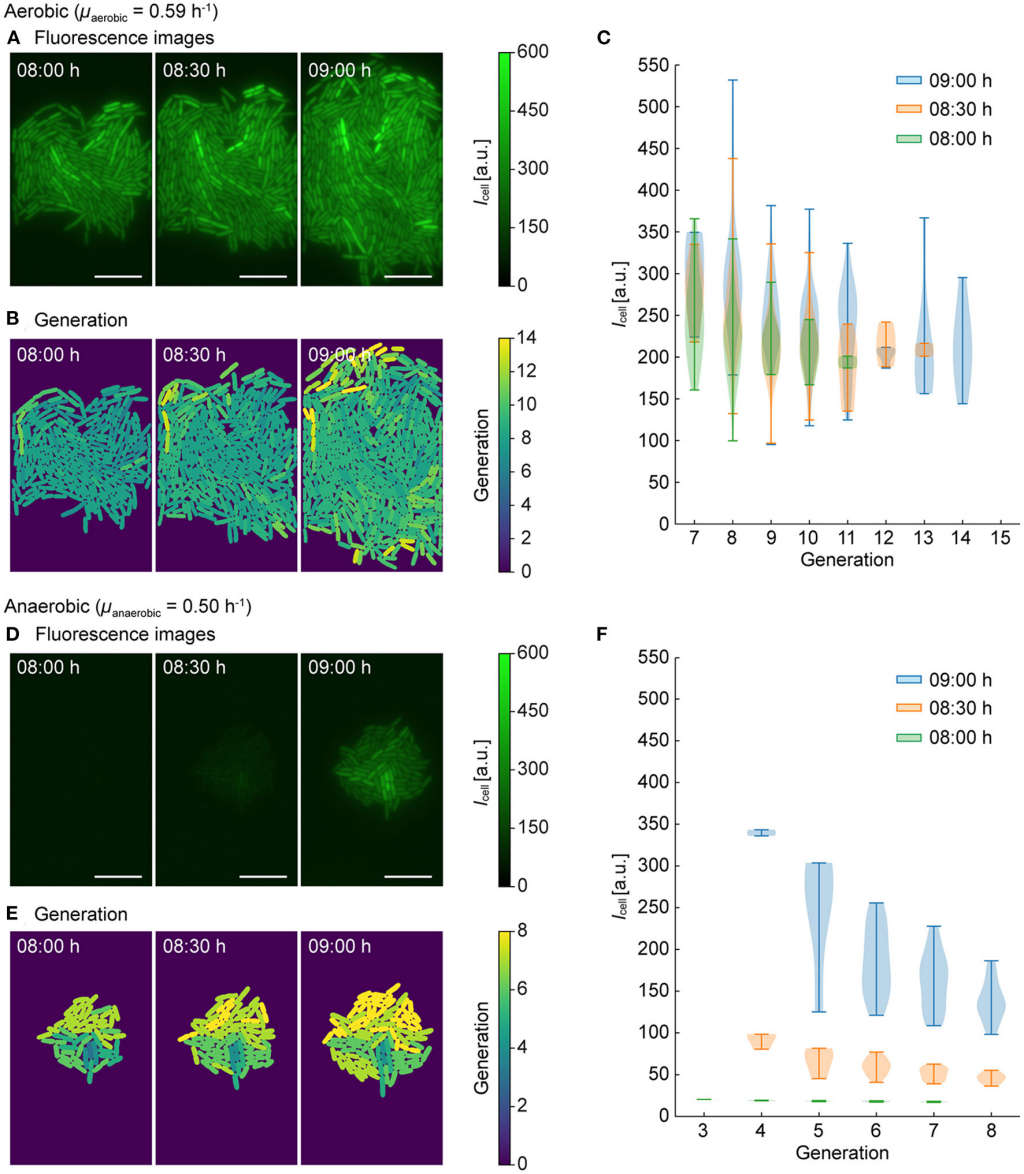
Single-cell tracking and analysis of fluorescence intensities (*I*_cell_) and generation numbers. One population was selected for **(A–C)** aerobic conditions and **(D–F)** anaerobic conditions with a switch to aerobic conditions. **(A, D)** Fluorescence intensity images at *t* = 08:00 h, 08:30 h, and 09:00 h (scale bars = 10 μm). **(B, E)** Generations number (i.e., number of division events detected) visualized by color coding. **(C, F)** *I*_cell_ and generations at three time points.

Under aerobic conditions, a growth rate of $\mu_{\text{aerobic}}=0.59\;{\text{h}}^{-1}$ was derived for the selected population. As shown in the fluorescence time-lapse images of Figure 5A, a pronounced and relatively homogeneous intracellular GFP fluorescence was detected at all time points. In addition to single cell fluorescence levels, image analysis provided each corresponding cell generation, with the results visualized in Figure 5B, where the color coding from blue to yellow indicates the generation. By plotting single-cell fluorescence *I*_cell_ over the generation (Figure 5C), correlations could be visualized. Here a slight tendency that cells with lower generation numbers (i.e., slow-growing cells) exhibited higher GFP fluorescence, while cells with higher generation numbers (i.e., fast-growing cells) exhibited lower GFP fluorescence.

Under anaerobic conditions ($\mu_{\text{anaerobic}}=0.50\;{\text{h}}^{-1}$) this effect was more prominent. As shown in the fluorescence images in Figure 5D, the GFP fluorescence was negligible before the switch at *t* = 08:00 h. After the switch from anaerobic to aerobic conditions, the GFP fluorescence of the cells rapidly increased as discussed before (*t* = 08:30 h, 09:00 h), but phenotypic population heterogeneity in terms of fluorescence intensity was more pronounced. The cells corresponding generation numbers are visualized in Figure 5E, also indicating a more pronounced heterogeneity in terms of single-cell growth rates. Moreover, the plot showing *I*_cell_ over generation number (Figure 5F), shows that intracellular GFP fluorescence decreases with the increasing division rate. This correlation is likely due to a higher dilution rate of intracellularly accumulated GFP during faster cell division.

#### Analysis at two different levels - population level and single-cell level

We performed the analysis of microorganisms at both, the population and single-cell level. The strength of our approach is that knowledge from different levels can be obtained from the same experiment. In addition to microfluidic monolayer cultivation, there are several widely used methods for microbial single-cell analysis, such as fluorescence-activated cell sorting and droplet confinement of single cells in a low volume of aqueous solution (Czechowska et al., 2008; Ishii et al., 2010; Joensson and Andersson Svahn, 2012). Although these are well-established technologies and provide data on very large numbers of cells, they are not suitable for spatiotemporal microbial analysis and therefore cannot deliver knowledge at the spatially resolved population level. However, considering that microbial heterogeneity of single cells can influence microbial behavior at the population level, observing microbial behavior at different levels - population and single-cell - and linking them would help to add more insight to microbiological research. The power of such an analysis becomes clearer when a microbial culture is performed in a structured oxygen environment.

## Conclusions

This paper describes a microfluidic cultivation setup for microbial single-cell analysis. In addition to environmental control by continuous media perfusion, it enables microbial cultures under oxygen control. To this end, a microfluidic cultivation chip was placed inside a simple and low-cost 3D-printed mini-incubator. A couple of mass flow controllers were used to deliver defined O_2_ / N_2_ gas mixtures, which in the present configuration allow to control oxygen supply in the range from 0% to 100% O_2_. The design allows a switching time of less than 100 minutes, which is currently only limited by the thickness of the gas permeable chip material PDMS. This thickness can be further reduced, but this requires more specialized chip assembly and handling.

Camera-based fluorescence lifetime imaging was used to monitor the O_2_-sensitive dye RTDP, which can be easily delivered as a sensor solution when pumped through the channels. Measuring the fluorescence lifetime of RTDP is more robust than measuring fluorescence intensity because the lifetime is independent of dye concentration. The RTDP sensor solution has a wide detection range and can be used as an oxygen sensor from 0% to 100% O_2_. The detection limit of RTDP as an oxygen sensor is reported to be around 0.5 μmol/L O_2_ (0% O_2_) (Berg et al., 2022). However, the present system is limited by the resolution and detection limit of the camera-based FLIM microscope setup, which we are currently investigating in more detail. Presumably, anaerobic cultures were performed when 100% N_2_ was supplied. It is assumed that we can operate the chip with at least 0.1 ppm O_2_, which was indirectly demonstrated by the incomplete chromophore maturation in the absence of oxygen when using GFP as an intracellular oxygen sensor.

Automated image analysis was used to extract cellular properties from large stacks of image data. All these tools are made available as open source. The cultivation setup can serve as an experiment-to-analysis system for various microbial single-cell analysis applications, such as the cultivation of micro-aerophilic and strictly anaerobic organisms whose single-cell behavior has not yet been thoroughly investigated. As iron solubility is dependent on oxygen concentration, studies of iron availability and its effect on cellular physiology are possible. A more advanced microfluidic configuration would also allow oxygen gradients and other patterns to be created. The setup is not limited to the control of oxygen and nitrogen gas, but can be adapted to other gases. We are currently using our device to grow photoautotrophic cyanobacteria while delivering defined CO_2_ gas mixtures.

## Data availability statement

The image-analysis tools presented in this study can be found in online repositories. The names of the repository/repositories and accession number(s) can be found below: https://github.com/JuBiotech/Supplement-to-Kasahara-et-al.-2023a.

## Author contributions

ML and DK designed the study. ML conducted the experiments. KK and ML performed the data analysis. KK, ML, and DK interpreted the results. JS, KR, and KN contributed to the automated image analysis. KK, TD, and DK wrote the paper. All authors contributed to the article and approved the submitted version.
