# Supplementary material for: Enabling oxygen-controlled microfluidic cultures for spatiotemporal microbial single-cell analysis
Source: Front Microbiol. 2023 Jun 20;14:1198170. doi: 10.3389/fmicb.2023.1198170 (PMC10318409; doi:10.3389/fmicb.2023.1198170)
Supplement: Supplementary file 1 [file Data_Sheet_1.PDF]

Supplementary Material

This PDF file includes:

Table S1. LB medium composition

Table S2. Defined M9 medium composition

Figure S1. Detailed design of the mini-incubator

Figure S2. FLIM calibration of RTDP and the O_2 step test at 25°C

1 SUPPLEMENTARY TABLES

Table S1. LB medium composition. All the chemicals were bought from Carl Roth, Germany. The pH was adjusted to 7.0 with NaOH. The solution was autoclaved at 121°C for 20 min, then stored at 4°C.

Component	Concentration [g/L]
Peptone	10
Yeast extract	5
NaCl	10

Table S2. Defined M9 medium composition. All the chemicals were bought from Carl Roth, Germany. The pH was adjusted to 7.0 with NaOH. The solution was autoclaved at 121°C for 20 min, then stored at 4°C.

Component	Concentration
Defined M9 medium [g/L]	
$Na_2HPO_4 \cdot 2\,H_2O$	7.528
KH_2PO_4	3.00
NaCl	0.50
NH_4Cl	1.00
$MgSO_4 \cdot 7\,H_2O$	0.24647
$CaCl_2 \cdot 2\,H_2O$	0.14701
Trace elements (1000X)	1 mL/L
Trace elements (1000X) [mM]	
$(NH_4)_6Mo_7O_{24} \cdot 4\,H_2O$	0.003
H_3BO_3	0.4
$CoCl_2 \cdot 6\,H_2O$	0.03
$CuSO_4 \cdot 5\,H_2O$	0.01
$MnCl_2 \cdot 3\,H_2O$	0.8
$ZnSO_4 \cdot 7\,H_2O$	0.01

2 SUPPLEMENTARY FIGURES

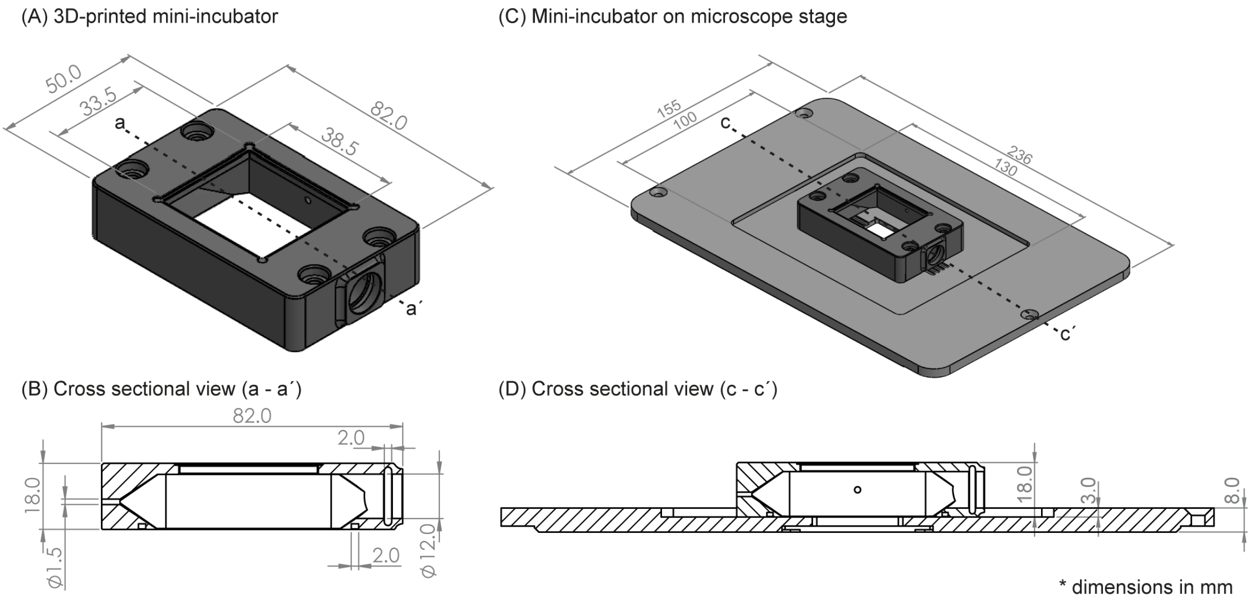

Figure S1. Detailed design of the mini-incubator. **(A)** Dimensions of the mini-incubator. **(B)** Cross sectional view of the mini-incubator (a -a ’). **(C)** Dimensions of the mini-incubator mounted on the microscope stage. **(D)** Cross-sectional view (c -c ’).

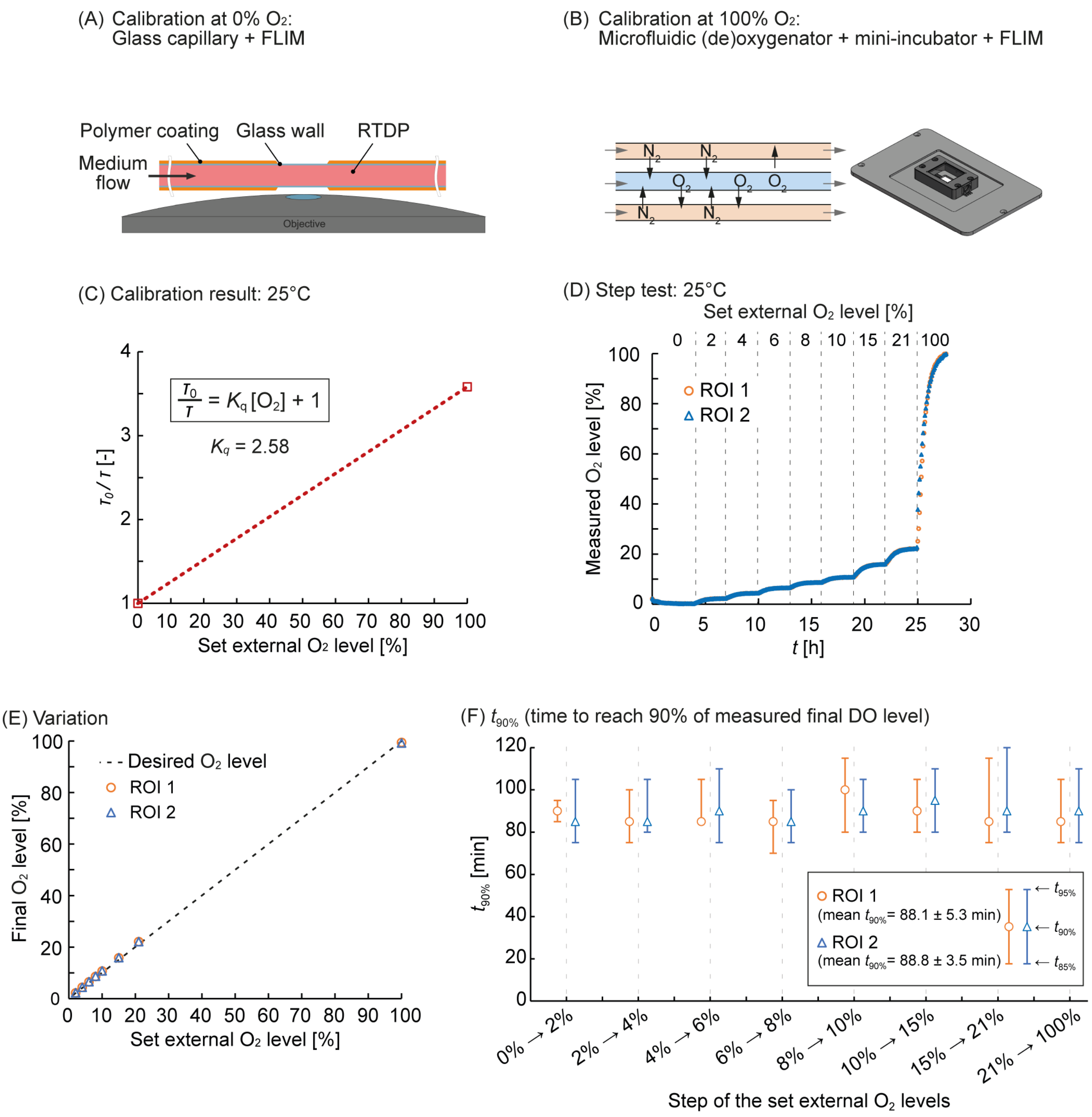

Figure S2. FLIM calibration of RTDP and the O_2 step test at 25°C. **(A)** Calibration at 0% O_2 level using the glass capillary. **(B)** Calibration at 100% O_2 level using the microfluidic (de)oxygenator and the mini-incubator. **(C)** Calibration plot of RTDP solution at 25°C, fitted with Stern-Volmer equation. **(D)** Oxygen concentration calculated from corresponding fluorescence lifetime at 25°C at ROI 1 and 2. **(E)** Measured final oxygen concentration and the desired O_2 concentration. **(F)** $t_{90\%}$ for each step of the set external O_2 levels. The middle markers indicate $t_{90\%}$, and the error bars mean $t_{85\%}$ and $t_{95\%}$.
